# A Self-Matting Waterborne Polyurethane Coating for PVC Artificial Leather

**DOI:** 10.3390/polym15010127

**Published:** 2022-12-28

**Authors:** Zhe Sun, Song Ren, Tong Wu, Jiating Wen, Jian Fang, Haojun Fan

**Affiliations:** 1 College of Textile and Clothing Engineering, Soochow University, Suzhou 215123, China; 2 Glorious Sun Guangdong School of Fashion, Huizhou University, Huizhou 516007, China; 3 Key Laboratory of Leather Chemistry and Engineering of Ministry of Education, Sichuan University, Chengdu 610065, China

**Keywords:** self-matting, waterborne polyurethane, structure–properties relationship, PVC artificial leather, application properties

## Abstract

A type of polyester-based self-matting waterborne polyurethane (ESMWPU) with an excellent matte effect of 0.8° and transmittance of 78.5% for PVC artificial leather was synthesized. The influence of synthesis parameters, including R value, crosslinking and hydrophilic group content, on coating gloss and transmittance was investigated. Meanwhile, the properties necessary for applying water-based resins to plasticized PVC were detailed. The results demonstrated that R value, crosslinking degree and hydrophilic group content synergistically decided the morphological changes of latex particles during their film-forming process in three aspects: particle stiffness, three-dimensional structure and particle size, respectively. With optimized parameters of R = 1.9, TMP = 2 wt% and DMPA = 1.75 wt%, ESMWPU latex particles stabilized their spherical shapes without collapsing in the film-forming process and created a rough surface, resulting in a matte effect. In terms of application performance, good wetting and adhesion for ESMWPU to a plasticized PVC surface was achieved with a 2 wt% leveling agent load. Moreover, due to the high cohesion energy of ester bonds and intermolecular hydrogen bonds, this type of polyester-based ESMWPU also depicted admirable thermal adhesion resistance. All aforementioned results distinctly demonstrate a feasible yet promising paradigm for applying ESMWPU on PVC artificial leather.

## 1. Introduction

Man-made leathers, fabricated by integrating polymer coating with fabric, have been widely applied in human lives, such as sofas, garments, bags, shoes, etc., because of their comparable properties to natural leathers [1,2,3]. According to the type of coating, man-made leathers can be divided into two categories: polyvinyl chloride (PVC) artificial leather and polyurethane (PU) synthetic leather [4,5]. In terms of coating structure, the artificial or synthetic leather coating generally consists of the surface effect layer, surface layer, intermediate foaming layer and adhesive layer [6]. Different types of coating play important roles in the overall physical properties of man-made leathers. Among them, the surface effect layer, which is mainly constructed from waterborne polyurethane (WPU), has received much attention due to its ability to give leathers various aesthetic and bandwagon effects [7,8].

With the transformation of people’s aesthetic concepts, leather products with low gloss have gradually become more popular with customers. Accordingly, WPU surface treatment agents with low gloss became the focus of intense research. Presently, the most normally used and economical way for preparing a matte treatment agent is by blending resins with various matting agents [9,10]. However, although the traditional physical method is convenient to realize, obstacles more or less still exist in application; for example, poor compatibility, heterogeneous appearance and low transmittance [11]. In view of this, great efforts have been focused on chemical extinction fields. In our previous work, a polyether-based self-matting WPU with a low gloss of 0.5° and high transmittance of 89.2% was achieved by balancing the contradiction between gloss and transmittance of coating [11]. In addition, Yong et al. [12,13] prepared a poly (tetramethylene oxide glycol)-based self-matting WPU which could be applied in leather finishing. 

However, although previously prepared self-matting WPU possesses excellent matte effects, they cannot be directly applied to PVC artificial leather. The reasons are as follows. On one hand, large amounts of plasticizers are essential in the manufacturing process of PVC coating to endow artificial leather with softness and flexibility. In this context, although PVC is a polar macromolecule, its surface energy changes greatly after being plasticized. Wetting and spreading properties of water-based resins on plasticized PVC need to be focused; on the other hand, due to the high polarity inherent in PVC and easy migration of plasticizer, the adhesion between WPU and plasticized PVC should not be ignored. Therefore, combining a water-based resin with the characteristics of plasticized PVC to develop a self-matting WPU treatment agent that can meet the requirements of artificial leather seems to be of particular importance.

Herein, the influence of synthesis factors, including R value, crosslinking degree and hydrophilic group content on optical properties of WPU was studied. After harmonizing all parameters, a type of polyester-based self-matting WPU (ESMWPU) with a gloss of 0.8° and transmittance of 78.5% together with admirable application properties was obtained, exhibiting high potential for practical application in surface treatment of PVC artificial leather. 

## 2. Experimental Section

### 2.1. Materials

Isophorone diisocyanate (IPDI), 2,2-Bis(hydroxymethyl) propionic acid (DMPA) and poly-1,3-butylene adipate glycol (PMA, M_n_ = 2000 g/mol) were provided by Dymatic Post Polymer Material Co. (Lishui, China). Trimethylolpropane (TMP), bismuth acid catalyst, triethylamine (TEA) and hydrazine hydrate were gained from Kelong Chemical Engineering Co. Ltd. (Chengdu, China). Leveling agent OFX-5211 was supplied by Dow Corning Co. (Midland, Michigan, USA). 

### 2.2. Synthesis of ESMWPU

**Figure 1:**
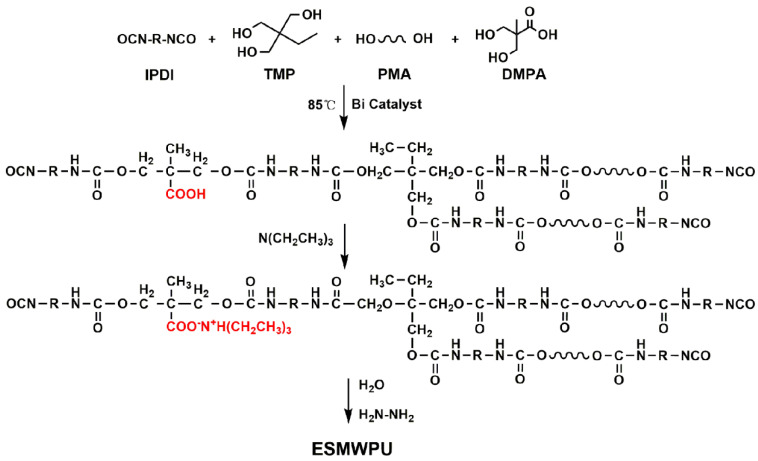
Synthesis route of ESMWPU.

The synthesis route of ESMWPU is shown in Figure 1.

First, stoichiometric IPDI, PMA, TMP, DMPA and bismuth acid catalyst were added into a three-neck flask equipped with a thermometer and stirrer. The reaction was conducted under N_2_ atmosphere at 85 °C for 5 h. Afterwards, the prepared prepolymer was cooled to 40 °C and neutralized by TEA. Finally, stoichiometric deionized water was added into the flask. An ESMWPU emulsion with known solid content was obtained. Postchain extension was executed with hydrazine hydrate solution. 

### 2.3. Preparation of WPU Films

WPU films with a thickness of 0.5 mm were attained by placing WPU emulsions on PTFE plates at ambient temperature for 3 days and then drying them for 24 h in an oven of 80 °C. All samples were placed in a desiccator at room temperature before characterization. 

### 2.4. Measurements

A Fourier transform infrared (FTIR) spectrum of ESMWPU film was gathered with an IS10 FTIR spectrometer (Thermo Scientific, Waltham, Massachusetts, United States) measuring attenuated total reflection (ATR) over the wavenumber range from 4000 to 400 cm^−1^. 

The optical properties of WPU films were tested using a WGT-S light transmittance and haze tester (Shanghai Jingke, China). First, the equipment was opened and preheated for 30 min. Then, films were fixed on the light hole, and the test button was clicked. The light transmittance of WPU films was averaged from three tests. 

The gloss of WPU-coated PVC artificial leather surfaces were tested with a gloss meter (REFO60, Germany). First, WPU was spread on PVC artificial leather surfaces by using linear rods. Then, the coated leathers were put into an oven at 130 °C for 2 min. All of these tests were conducted with an incidence angle of 60°. 

The surface morphologies of the coatings were observed via scanning electron microscope (SEM) (JSM-7500F, Japan). The magnification used was 1000 times.

Three-dimensional morphological observation of the coatings was carried out with an atomic force microscope (AFM) (SPM-9500, Japan). The scanning rate applied was 1 Hz. 

The ESMWPU dispersions’ particle sizes were measured using a Mastersizer 2000 (Malvern Instruments). 

Surface energy of solid film was calculated by the equation of Zhu et al. [14,15] as follows:

(1) $$\gamma_{SL}=\frac{\gamma_{L}}{2}\left(\sqrt{1+\sin^{2}\theta }-\cos\theta \right)\;0\leq \theta \leq 180{}^{\circ}$$

(2) $$\gamma_{S}=\frac{\gamma_{L}}{2}\left(\sqrt{1+\sin^{2}\theta }+\cos\theta \right)\;0\leq \theta \leq 180{}^{\circ}$$

where $\gamma_{SL}$, $\gamma_{L}$, $\gamma_{S}$ and *θ* are solid-liquid interfacial tension, surface tension of liquid, surface energy of solid film and contact angle, respectively. Further, the wetting and spreading properties were evaluated with following equation:

(3) Sc=–ΔG/A=γS−γL−γSL

where $S_{C}$ is the spreading coefficient of ESMWPU emulsion on PVC artificial leather. 

An adhesion test was carried out according to the ISO 2409: 1992 standard (Paints and varnishes-Cross cut test for films). 

A thermal adhesion resistance test was conducted according to the GB/T 8948-2008 standard (Polyvinyl chloride artificial leather). First, ESMWPU-coated PVC artificial leather was folded face-to-face. Then, the folded leather was pressed with a 5 kg weight and transferred to an oven at 85 °C for 12 h. The thermal adhesion property was evaluated by examining the difficulty level of peeling the folded face-to-face coating. 

A wear resistance test was carried out according to the ASTM D 4060 (Standard Test Method for Abrasion Resistance of Organic Coatings by the Taber Abraser). The auxiliary mass was 1 kg.

## 3. Results and Discussion

### 3.1. Characterization of ESMWPU

**Figure 2:**
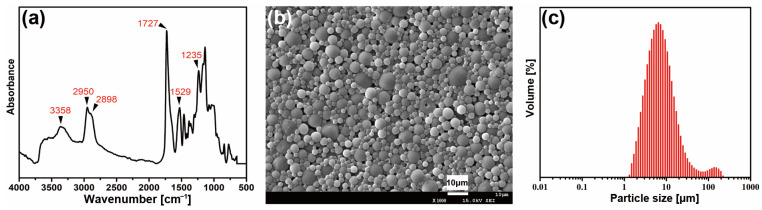
(**a**) FTIR of ESMWPU, (**b**) surface morphology of ESMWPU film and (**c**) latex particles’ size in ESMWPU emulsion.

The FTIR and surface morphology of ESMWPU film and the size of latex particles in ESMWPU emulsionare depicted in Figure 2. 

**Figure 3:**
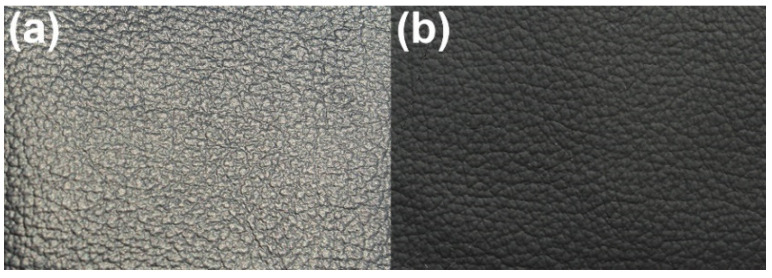
Matting effect of ESMWPU: (**a**) Original PVC artificial leather, (**b**) ESMWPU-coated PVC artificial leather.

As shown in Figure 2a, the characteristic absorption bands at 2270 cm^−1^ and 3500 cm^−1^, referred to as the -N=C=O and -OH groups, disappear, indicating that all isocyanate and hydroxyl groups were completely reacted in this system. As for the ESMWPU resin, the broad band at 3358 cm^−1^ can be attributed to the N-H stretching vibration. The bands at 2950 cm^−1^ and 2898 cm^−1^ are attributed to the C-H stretching vibration. PMA exhibits a strong absorption band at 1727 cm^−1^ belonging to the stretching vibration of C=O. The 1529 cm^−1^ and 1235 cm^−1^ bands are assigned to C-N and N-CO-O/C-O-C, respectively. Meanwhile, it can be seen that the ESMWPU film surface presented an extensive accumulation of spherical particles instead of a flat surface (Figure 2b). This phenomenon can be attributed to elaborately designed latex particles with a large size of 7.2 μm on average (Figure 2c) as well as the stiffness of particles as endowed by synthesis parameters. Specifically, higher R value and appropriate cross-linking endow polyurethane macromolecules with their three-dimensional structure and increased hard segment density. In the film-forming process, the shape of latex particles was well-stabilized instead of collapsing, thus forming a rough surface. As a result, when incidental light shined on the surface, an intense diffuse reflection occured rather than specular reflection, and a matting effect was achieved. The intuitive matting effect obtained by coating PVC artificial leather with ESMWPU is shown in Figure 3. 

### 3.2. Relationship between Synthesis Parameters and Coating Properties

#### 3.2.1. Effect of *R* Value on Surface Morphology and Optical Properties

R value, as a molar ratio between isocyanate (-NCO) and hydroxyl (-OH) groups, is an important parameter for WPU preparation. Herein, variations of surface morphology and the optical properties of WPU coatings with different R values were investigated.

**Figure 4:**
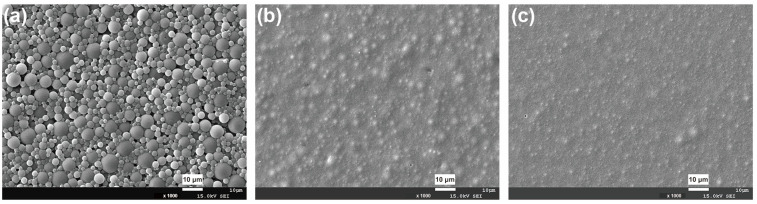
Effect of R value on surface morphology of WPU coatings: (**a**) R = 1.9, (**b**) R = 2.1 and (**c**) R = 2.3.

As shown in Figure 4, with the increase in R value, the microsphere structure of the coating surface progressively disappeared, and it transformed from a rough surface to a smooth surface. Accordingly, both coating gloss and film transmittance increased as well (Table 1). These phenomena can be attributed to the fact that a higher R value causes a decrease in molecular weight and a lower particle size, which in turn leads to a decline in surface roughness. The reduced surface roughness brings about a decrease in diffuse reflection intensity from incidental light on the coating surface, thus causing an increase in gloss and transmittance. 

#### 3.2.2. Effect of TMP Content on Surface Morphology and Optical Properties

**Figure 5:**
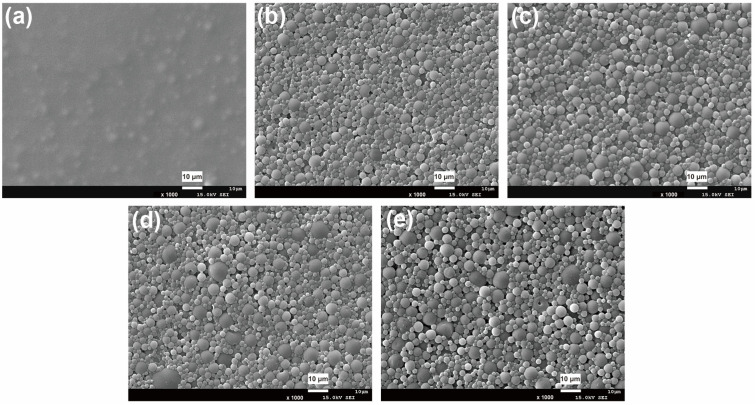
Effect of TMP content on surface morphology of WPU coatings: (**a**) 0 wt%, (**b**) 0.5 wt%, (**c**) 1.0 wt%, (**d**) 1.5 wt% and (**e**) 2.0 wt%.

Crosslinking always endows a polymer with a three-dimensional structure. Figure 5 presents the SEM images of a WPU coating surface before and after crosslinking. It can be seen that, when no crosslinker is added, the coating surface is relatively smooth. However, after incorporating TMP, the coating surface became rough and was accompanied by a remarkable decline in gloss (Table 1). The results indicate that TMP is essential for the construction of matte WPU. 

**Figure 6:**
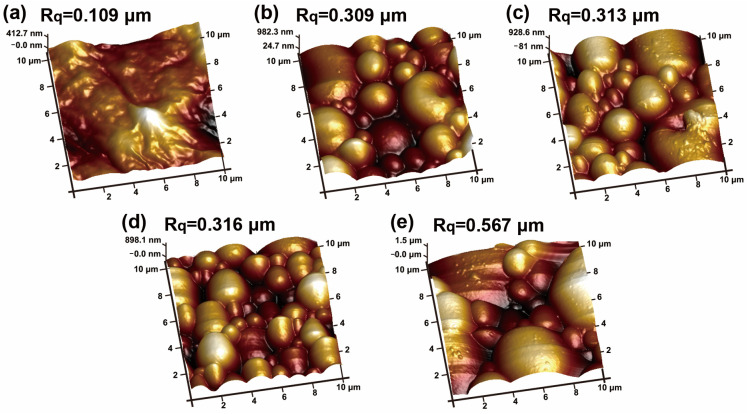
Effect of TMP content on surface roughness of WPU coatings: (**a**) 0 wt%, (**b**) 0.5 wt%, (**c**) 1.0 wt%, (**d**) 1.5 wt% and (**e**) 2.0 wt%.

Further, the effect of different crosslinker content levels on the surface roughness of WPU coatings is depicted in Figure 6. As shown in the figure, when no crosslinker is added, the coating surface roughness (R_q_) is only 0.109 μm and the corresponding coating gloss is as high as 5.1°. After adding 0.5, 1.0, 1.5 and 2.0 wt% TMP, the R_q_ increased to 0.309, 0.313, 0.316 and 0.567 μm, respectively, which in turn manifested as a decrease in coating gloss (Table 1). 

#### 3.2.3. Effect of DMPA Content on Surface Morphology and Optical Properties

DMPA is the most commonly used hydrophilic chain extender for preparing anionic WPU. The WPU emulsion particle size and hydrophilic group content are closely related. 

**Figure 7:**
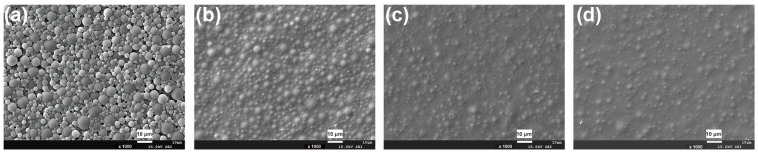
Effect of DMPA on surface morphology of WPU coatings: (**a**) 1.75 wt%, (**b**) 1.90 wt%, (**c**) 2.05 wt% and (**d**) 2.20 wt%.

The effect of DMPA content on the surface morphology of WPU coating is shown in Figure 7. It can be seen that as DMPA wt% increased from 1.75 to 2.20 wt%, the coating’s microroughness gradually decreased. When DMPA content was 1.75 wt%, the microsphere structure was obvious; whereas when DMPA wt% > 1.90, the microscopic convex structure was difficult to observe. This phenomenon can be attributed to the decreasing emulsion particle size caused by increasing DMPA content. Table 1 shows the effect of DMPA wt% on coating transmittance and gloss. A rough surface and a low coating gloss of 0.8° were achieved with a DMPA content of 1.75 wt%. 

### 3.3. Application of ESMWPU on PVC Artificial Leather

#### 3.3.1. Wetting Properties

Pristine PVC materials are always too brittle for any practical purpose. Hence, to accommodate the softness requirements of artificial leather, a large amount (40–120 wt%) of plasticizer must be added to PVC resins to bestow their coating with flexibility and bendability [16]. These lubricant-like plasticizers inadvertently lead to a variation in coating surface energy. Therefore, when ESMWPU is applied as surface treatment agent for PVC artificial leather, it is necessary to investigate its wetting and spreading properties. Usually, lower contact angles and interfacial tension imply better wetting ability [17].

**Figure 8:**
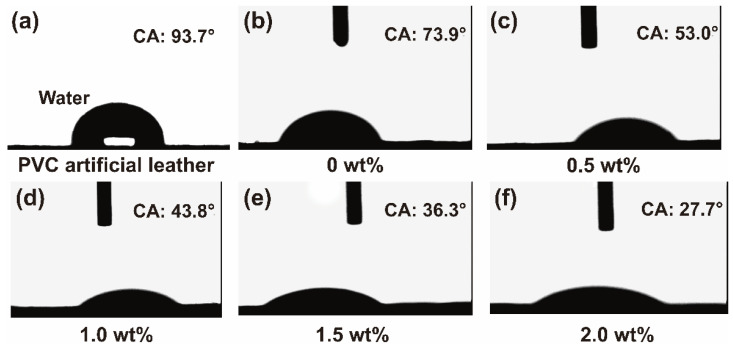
(**a**) WCA of PVC artificial leather surface; (**b**–**f**) effect of leveling agent on the CA of ESMWPU on the surface of PVC artificial leather.

Figure 8a presents the water contact angle (WCA) of a PVC artificial leather surface, and the corresponding surface energy (48.5 mJ/m^2^) can be calculated with the Zhu et al. equation. Based on this, the contact angles (Cas) of ESMWPU emulsions on PVC artificial leather with different leveling agent dosages were tested (Figure 8b–f). The results show that the CA of original ESMWPU on a plasticized PVC surface was 73.9°. With the addition of a leveling agent, CA gradually decreased to 27.7°, representing excellent wetting and spreading properties. 

To further demonstrate wettability, two vital parameters, interfacial tension and spreading coefficient (*Sc*), between ESMWPU and plasticized PVC substrates were calculated. As shown in Table 2, both the surface and interfacial tension show an obvious decrease with an increasing amount of leveling agent. Accordingly, the S_C_ shows a significant increase. When leveling agent content is 2 wt%, the S_C_ can reach up to 24.1 mJ/m^2^, which can meet the requirement of three-plate printing for artificial leather surface treatment. 

#### 3.3.2. Adhesion

**Figure 9:**
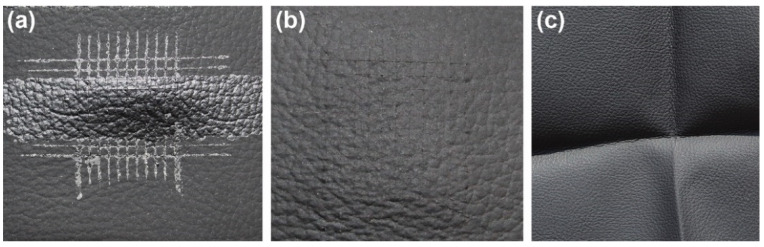
The adhesion of (**a**) polyether and (**b**) polyester-based self-matting WPU on plasticized PVC; (**c**) The thermal-adhesion resistance of ESMWPU coating.

Adhesion is a key parameter for coatings, especially for plasticized PVC artificial leather. The migration of plasticizers is detrimental to the adhesion between PVC and the surface treatment agent. Thus, assuming the ESMWPU is well-wetted and spread, it is necessary to evaluate the adhesion of ESMWPU on a PVC surface. Figure 9a,b depicts the comparison of the adhesion of ESMWPU and polyether-based matte WPU as prepared in our previous work [11]. The results show that the polyether-based WPU is easily peeled off during the cross-cut test, whereas the polyester-based ESMWPU remains intact. This is because the plasticized PVC exhibits high polarity due to the inherent polarity of the PVC molecules and the addition of polar plasticizers. According to the principle of similar compatibility, the polar polyester-based ESMWPU shows more affinity with plasticized PVC. According to ISO 2409: 1992, the adhesion grade can be rated as level 0. 

#### 3.3.3. Resistance to Thermal Adhesion

The thermal-adhesion resistance property of coating is another important index to evaluate the performance of artificial leather. Our tests show that folded artificial leather was easily peeled off with gentle force even after a long period of hot-pressing for 12 h; meanwhile, no damage or breakages © on the coating surface or the fold area. (Figure 9c) This demonstrates the excellent thermal adhesion resistance of ESMWPU coating, which can be evaluated as grade 5 according to GB/T 8948-2008. This satisfactory exhibition can be attributed to the ester bonds on PMA polyols. Their high cohesive energy, internal rotational barriers and the hydrogen bonds formed with -NHCOO- make PU macromolecular chains hard to move and migrate during the hot-pressing process, which in turn produces excellent resistance to thermal adhesion. 

#### 3.3.4. Wear Resistance

**Figure 10:**
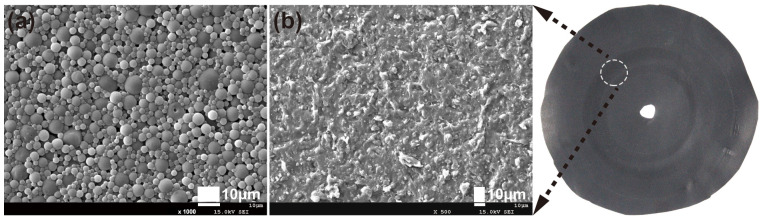
SEM micrographs of ESMWPU coating (**a**) before and (**b**) after 300 abrasions.

Wear resistance is another important parameter in the evaluation of artificial leather coating performance. After testing, the as-prepared pristine ESMWPU coating endured 300 abrasions under 1 kg auxiliary mass, which meets the basic requirements. (Figure 10) However, for areas that require higher wear resistance, the wear resistance of ESMWPU must be further improved. Preservation of the unique surface microsphere structure (surface roughness) of ESMWPU will also pose a challenge. More systematic studies will be conducted in our future work. 

## 4. Conclusions

A type of polyester-based self-matting waterborne polyurethane (ESMWPU) for surface treatment PVC artificial leather was synthesized, and associations between synthesis factors and the optical properties of WPU were found. The results demonstrated that R value, crosslinking and hydrophilic group content synergistically affected the film-forming morphology of latex particles, which in turn decided the roughness and gloss of coating surface: R value adjusted the hardness of latex particles by changing their hard segment content; cross-linking, which endowed latex particles morphological stability in the film-forming process by giving polyurethane macromolecules a three-dimensional structure, is essential for preparing low-gloss coating; hydrophilic group content impacted the matte effect by regulating particle size. When DMPA = 1.75 wt%, a large particle size of 7–8 μm was obtained. By maintaining synergy with the previous parameters, the spherical shapes of latex particles were well-maintained during the film-forming process, and the desired surface roughness and a subsequently excellent matte effect were achieved. In applications, the leveling agent efficiently decreased the surface tension of ESMWPU and ensured effective wetting and spreading on plasticized PVC. Meanwhile, the high cohesion energy of ester bonds and intermolecular hydrogen bonds formed with -NOCOO- endowed ESMWPU bestowed admirable thermal-adhesion resistance and adhesion to plasticized PVC.

## Figures and Tables

**Table 1:** Effect of R value, TMP and DMPA content on gloss and transmittance.

Synthesis Parameters	Values	Gloss (°)	Transmittance (%)
R value	1.9	0.8	78.5
	2.1	4.0	88.5
	2.3	4.9	89.0
TMP content (wt%)	0	5.1	88.0
	0.5	1.1	80.8
	1.0	1.0	80.0
	1.5	0.9	79.1
	2.0	0.8	78.5
DMPA content (wt%)	1.75	0.8	78.5
	1.90	1.7	85.5
	2.05	3.2	88.5
	2.20	4.8	88.8

**Table 2:** Effect of leveling agent content on contact angle, *γ_SL_*, *γ_L_* and *S_c_*.

Leveling Agent (wt%)	Contact Angle (°)	γ_SL_ (mJ/m^2^)	γ_L_ (mN/m)	Sc (mJ/m^2^)
0	73.9	25.5	46.0	−23
0.5	53.0	7.5	22.1	18.9
1.0	43.8	5.4	22.0	21.1
1.5	36.3	3.9	22.0	22.6
2.0	27.7	2.4	22.0	24.1

## Data Availability

The data presented in this study are available on request from the corresponding author.
